# Self-diagnosis of malaria by travellers: a cohort study on the use of malaria rapid diagnostic tests provided by a Swiss travel clinic

**DOI:** 10.1186/s12936-017-2079-2

**Published:** 2017-10-28

**Authors:** Delphine Berthod, Jacynthe Rochat, Rachel Voumard, Laurence Rochat, Blaise Genton, Valérie D’Acremont

**Affiliations:** 1 Travel Clinic, Department of Ambulatory Care and Community Medicine, University of Lausanne, Lausanne, Switzerland; 2 Infectious Diseases Service, Department of Medicine, University Hospital and University of Lausanne, Lausanne, Switzerland; 3 Swiss Tropical and Public Health Institute, University of Basel, Basel, Switzerland

**Keywords:** Malaria rapid diagnostic test, Traveller, Self-diagnosis, Training run

## Abstract

**Background:**

The WHO recommends that all suspect malaria cases be tested before receiving treatment. Rapid diagnostic tests (RDT) for malaria can be performed reliably by community health workers with no formal medical background and thus, RDTs could also be provided to travellers for self-diagnosis during visits to endemic regions.

**Methods:**

RDTs were proposed during pre-travel consultations to pre-defined categories of travellers. A training run on their own blood was performed and, if carried out correctly, the traveller was given a written procedure on how to perform the test and act on its result. The travellers were then proposed to buy a malaria RDT kit and were interviewed upon their return.

**Results:**

From February 2012 to February 2017, 744 travellers were proposed RDTs and 692 performed the training run (one could not complete it due to a hand tremor). Among the 691 subjects included, 69% travelled to moderate- or low-risk areas of malaria, 18% to high-risk areas and 13% to mixed-risk areas. The two most frequent categories of travellers to whom RDTs were proposed were long-term travellers (69%) and those travelling to remote areas (57%). 543 travellers (79%) were interviewed upon return. During their trip, 17% (91/543) had a medical problem with fever and 12% (65/543) without fever. Among 91 febrile patients, 57% (52/91) performed an RDT, 22% (20/91) consulted immediately without using the test, and 21% (19/91) did neither. Four RDTs (4/52; 8%) were positive: 2 in low-risk and 2 in high-risk areas (0.7% attack rate of self-documented malaria). Two travellers could not perform the test correctly and attended a facility or took standby emergency treatment. Four travellers with negative results repeated the test after 24 h; all were still negative. Carrying RDTs made travellers feel more secure, especially when travelling with children.

**Conclusions:**

1/6 travellers experienced fever and 4/5 of those reacted appropriately: more than half used RDTs and a quarter consulted immediately. Four travellers (including 2 from low-risk areas) diagnosed themselves with malaria and self-treated successfully. This strategy allows prompt treatment for malaria in high-risk groups and may avoid over-diagnosis (and subsequent inappropriate treatment) of malaria on-site.

**Electronic supplementary material:**

The online version of this article (doi:10.1186/s12936-017-2079-2) contains supplementary material, which is available to authorized users.

## Background

Before prescribing anti-malarial therapy in any setting, the WHO recommends a confirmation of parasitaemia by microscopy or malaria antigen-detecting immunochromatographic rapid diagnostic test (RDT) [1]. Early, accurate diagnosis of malaria is also key to prompt treatment which is especially important among non-immune travellers to endemic countries, who often suffer more severe complications [2]. A meta-analysis of RDTs for *Plasmodium falciparum* among travellers in 2005 reported 88–99% sensitivity and 95–100% specificity compared to microscopy, with an excellent negative likelihood ratio (0.05) [3]. Recent studies in non-endemic and endemic settings using PCR as reference test showed that sensitivity of RDTs is even higher than standard (but not necessarily expert) microscopy [4].

The use of RDTs by travellers has been controversial. In 1999, Trachsler et al. reported that a high proportion (14%) of travellers in a pre-travel setting was not able to interpret dipstick RDTs correctly despite receiving written instructions and/or oral explanations. The authors thus proposed to perform a training run [5]. Funk et al. reported high levels of false-negative interpretation by travellers with MalaQuick^®^ and ParaSight F^®^ despite an information leaflet [6]. In the same year, Jelinek et al. showed that 31% of febrile travellers in Kenya failed to perform the dipstick test [7]. Self-testing by ill travellers has also been studied by Whitty et al. with 9% of travellers not being able to obtain a valid result; they concluded that clearer instructions were essential [8].

On the other hand, several studies have now shown that, with appropriate instructions, RDTs could be used reliably by persons without formal medical training, such as oil field employees [9] and community health workers [10]. In an extensive review on the use of RDT in travel medicine, Maltha et al. concluded that RDTs for self-diagnosis may be useful for the traveller when comprehensive instructions and a training programme are guaranteed, but that further studies were needed under field settings [11].

The primary aim of this study was to explore the use of RDTs for self-diagnosis among selected travellers after being provided with practical, oral and written instructions during a pre-travel consultation. A second objective was to investigate whether these travellers would consider taking RDTs again for another similar trip.

## Methods

The protocol was approved by the ethics review board of the University of Lausanne, Switzerland. The study was conducted at the Travel Clinic of the University hospital of Lausanne from February 2012 to February 2017. RDTs were primarily proposed to travellers planning to visit moderate- or low-risk malaria areas (i.e. to whom the Swiss guidelines on malaria prevention [12] recommend the use of standby emergency treatment (SBET) rather than chemoprophylaxis). Categories of adult travellers likely to benefit most from carrying RDTs were selected as those at higher risk of not having access to rapid/accurate diagnosis on-site, and those known to underuse personal protective measures and to poorly adhere to recommendations [13–15], namely chemoprophylaxis [16]). These categories were: (a) humanitarian workers, (b) short-stay frequent travellers (≥ 3 trips of ≤ 7 days/year), (c) long-term travellers (> 3 months), (d) travellers to remote areas (> 24 h delay to access medical care), (e) travellers not willing to take malaria chemoprophylaxis although recommended by the Swiss guidelines. Health care professionals were also included because of their potential ability to perform tests as well as travellers willing to take the test even if not belonging to one of the above-mentioned categories.

Travellers attending pre-travel consultation and pertaining to one of these categories were proposed to participate in the study. Demographic data were recorded. At the end of the pre-travel consultation, and if they had given consent, travellers were explained how to perform the test using graphic and written instructions that detailed the procedure (see Additional file 1). The traveller’s ability to perform the test on her/himself (including a real finger-prick blood draw) was assessed in front of the health professional. If this training run using their blood was done correctly, travellers were asked to look at pictures of positive, negative and invalid tests and to interpret the results. Oral instruction was then given on how they should react in case of febrile illness (≥ 37.5 °C axillary temperature or clear subjective sensation of fever) during the trip. If there was no possibility to attend a medical facility within 24 h, they were told to perform an RDT. In case of positive test, they were recommended to immediately take SBET using artemether/lumefantrine or atovaquone/proguanil, and to seek medical advice to exclude severe malaria. In case of a negative result, they were told to go to a health facility to look for an alternate diagnosis, and to repeat the RDT after 24 h if fever persisted (in order not to miss the beginning of a malaria episode with low parasite density), as has been recommended and proven safe for febrile travellers returning from the tropics [17]. In case of an invalid result, they were told to repeat the test immediately using a new cassette. Travellers were finally provided with a prescription for a kit of 2 RDTs and related material and for a SBET. The test proposed was the rapid antigen capture assay ICT Combo^®^ ML02^®^ (ICT Diagnostics, South Africa) up to July 2015 and then, due to procurement constraints, SD Bioline^®^ Pf/Pan^®^. Both tests showed an adequate panel detection score (PDS) for *P. falciparum* in the WHO Round 5 of Product Testing of Malaria RDTs [18]. Tests combining HRP and a panmalarial antigen detection were used so that *P. vivax* could also be detected and to overcome the problem of HRP2 deletion found in some regions [19]. From December 2014, the pipette included in the ICT Combo test^®^ was changed for an inverted cup because a difficulty in transferring blood to the device had been noticed. The SD Bioline^®^ contained an inverted cup. The 2 RDTs kit cost 20 Swiss francs.

Upon return, travellers were contacted via phone or email by a study nurse who recorded detailed information on destinations and dates of the trip, medical issues arising during the trip, use of the purchased RDTs and the traveller’s wish to take RDTs again for another, similar trip. If used, the results of the RDT were also recorded along with information on difficulties in performing the test, and the traveller’s reaction to the result (such as seeking on-site consultation). All data were collected in Microsoft Excel and analyzed using *Stata (version 14).* A traveller was considered to have had a malaria episode if they had had a feeling of fever and/or an elevated temperature and a positive RDT result.

## Results

Between February 2012 and February 2017 RDTs were proposed to 744 travellers. Reasons for refusal were recorded during a period of 2 years (between 01.02.2013 and 28.02.2015) during which 479 travellers were seen. The main reasons among the 52/479 (11%) who were not interested in taking RDTs were the preference of attending a medical facility on-site in case of fever, and a reluctance to prick themselves (Table 1).

**Table 1:** Reasons mentioned by travellers for not being interested in taking RDTs

Reasons for not being interested to take RDTs (multiple entries possible)	Total N = 52 (%)
Sufficient accessibility to on-site medical care	24 (46)
Reluctance to prick themselves	20 (38)
No interest, useless measure	11 (21)
Insufficient money to buy the test	8 (15)
Reluctance to carry the test	3 (6)
Risk too low to justify measure	2 (4)

**Fig. 1:**
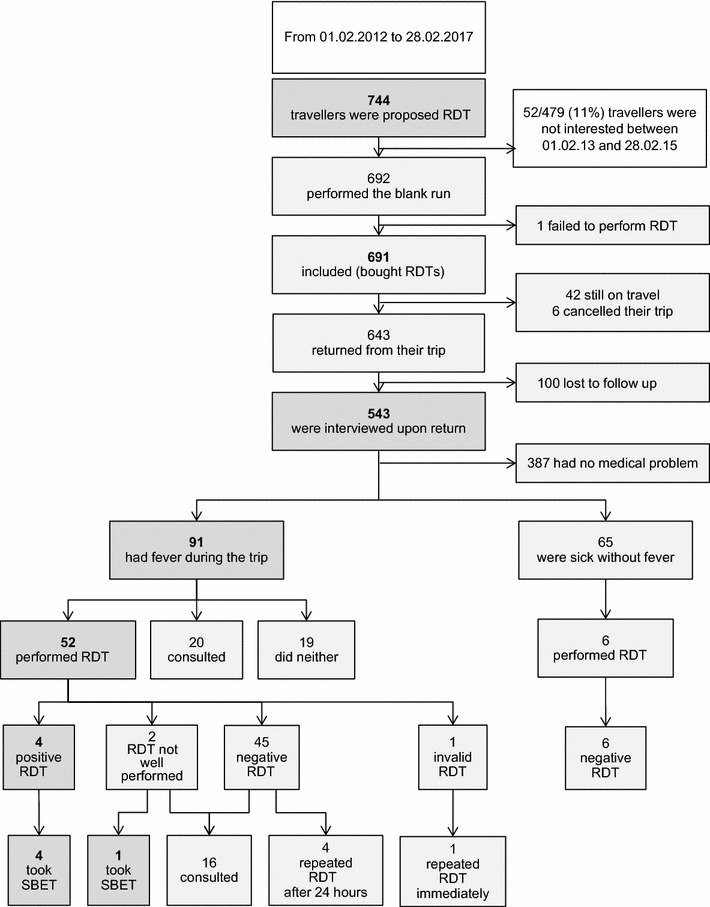
Flowchart of travellers included in the study

692 travellers were enrolled and performed the training run (Fig. 1). One traveller failed to perform the training run because of shaking hands. 691 travellers were thus followed for evaluation. Median age of travellers was 33 years (range 20 months to 74 years); 54% were females. 69% were travelling to malaria moderate- to low-risk areas, 18% to high-risk and 13% to both. The categories of travellers to whom RDT were proposed are shown in Table 2.

**Table 2:** Categories of travellers which were proposed RDT travel kits

Travellers’ category (multiple entries possible)	Total N = 691 n (%)
Long-term traveller (> 3 months)	478 (69%)
Traveller to remote areas	397 (57%)
Traveller’s request	117 (17%)
Health care professional	97 (14%)
Humanitarian worker	77 (11%)
Refusal to take chemoprophylaxis^a^	68 (10%)
Short-stay frequent traveller	30 (4%)
Others	32 (5%)
Travellers with children	12
Pregnancy (known/desired)	8
Expatriate	8
Visiting friends and relatives (VFR)	2
Fear of serious drug interaction^b^	2
Other reason, not specified	20

^a^Despite visiting a high malaria risk area

^b^Between their routine medication (antidepressant and antiepileptic) and anti-malarials

Data on the actual use of RDT during the trip was available for 543 travellers who could be interviewed upon return (78%). Of the 148 who were not interviewed, 6 had their trip cancelled, 100 were lost to follow-up and 42 were still travelling at the time the study ended. The median length of stay abroad was 18 weeks (range 5 days to 4 years). 71% had travelled to a moderate- or low-risk area, 20% to a high-risk area and 9% to destinations including both types of risk areas. 71% (387/543) had no medical problem during their trip; 17% (91/543) had a medical problem with fever, and 12% (65/543) without fever.

Among the 91 febrile travellers, 64% had travelled to a moderate- or low-risk area. Seventy-two of those (79%) strictly followed the recommendations provided in the written procedure: 52 (57%) performed an RDT (on a traveller’s child in 7 cases and on a febrile relative or friend in 6 cases), and 20 (22%) decided to directly attend a health facility, and thus, did not use the RDT. Diagnoses assigned to those attending health facilities were: diarrhoea (11), typhoid fever or salmonellosis (2), ORL infection (2), dengue fever (1), pneumonia (1), cellulitis (1), urinary tract infection (1); one was diagnosed as malaria. This female traveller attended an outpatient clinic in the Democratic Republic of Congo on the first day of her fever, where a rapid diagnostic test (provided by the clinic) and a blood slide were negative. Two days later she attended again and both were positive with 3% parasitaemia. She recovered after quinine and artemether-lumefantrine treatment. The traveller had stopped her prophylaxis of doxycycline 2 months prior to this episode following recommendations of fellow expats.

Nineteen travellers (20%) neither used their RDTs nor sought medical advice, because of concurrent transient diarrhoea (17) providing an alternate diagnosis or because the duration of fever was limited to a few hours (2). Among the 52 febrile travellers who performed RDTs, 4 (8%) had a positive result; 2 among travellers to low-risk areas (Haiti and Indonesia) and 2 to high-risk areas (Southern Sudan and Cameroon). All four malaria cases took adequate SBET and recovered. One attended a local clinic thereafter, while the 3 others did not. Among those, one was unsure about the result because he saw a faint line but took the SBET as instructed. He repeated the test after 24 h and found again a positive result.

Fifty-one RDT results were negative among 21 travellers to high-risk and 30 to moderate- to low-risk areas. 6 were performed in non-febrile travellers. Fifteen travellers sought medical advice afterwards while 36 did not. Four travellers repeated the test after 24 h, and the result was, again, negative. The others did not because they were fever-free by that time. One test was invalid; the test was immediately repeated and the result was negative. In two cases, the test was not properly performed. One traveller to Ecuador read the result after several hours because he had fallen asleep. As the test seemed positive, he immediately went to hospital, where he presented with no fever and was sent back home with neither test nor treatment. On the following day he attended a clinic specialized in malaria because of headache. A rapid diagnostic test was negative and the traveller left without anti-malarial drugs. Another traveller in Cameroon had lost the instruction leaflet and had difficulty transferring blood to the device. As he was not sure how to interpret the result, he decided to take the SBET.

When asked upon return, 90% (488/543) of the travellers stated that they would take RDTs again for a future, similar trip while 10% (55/543) would not, without statistically significant difference between those who had fever or not during their trip (11 and 10% respectively; p = 0.725) or those having used a RDT or not (9 and 10% respectively; p = 0.714).

The reasons for intending to take or not take RDT for a future trip are presented in Table 3.

**Table 3:** Travellers’ reasons for intending to take or not take RDTs for a future similar trip (open question analysed and categorized)

Reasons for taking RDT again (multiple entries possible) Total N = 488	Reasons for NOT taking RDT again (multiple entries possible) Total N = 55
220 “Reassuring”	9 “Good health infrastructures on-site”^a^
53 “To avoid unnecessary treatment”	7 “I am not at risk”, “I avoid risk areas”
28 “Especially useful when travelling with children”	5 “Prophylaxis will be enough”
11 “Convenient, practical”	5 “Useless”
8 “Help to react more quickly, autonomy”	4 “Protection against mosquito bites is enough”
1 “I will take prophylaxis and RDT, even in a low-risk area, because I already experienced malaria”	1 “Thought of being able to distinguish malaria symptoms from other origin”
167 No reason mentioned	24 No reason mentioned

^a^But one would still take RDT if travelling with children

## Discussion

One out of six travellers (16.7%) experienced fever during their trip, which is much higher than the 3% reported among the average American traveller [20]. Most of these febrile travellers (4 out of 5) reacted properly by either performing an RDT (more than half), or attending a health facility (almost a quarter). This rate is satisfactory, comparing to previous studies. For example, in 1995, Schlagenhauf et al. reported that 2 out of 3 travellers to low-risk areas, who had received pre-travel advice, failed to seek prompt medical advice despite having fever [21]. Further, in a German prospective study, only 34% of febrile travellers who received SBET in a pre-travel consultation either sought medical care [16/167 (10%)] or took SBET [(40/167 (24%)] [22].

A recent study analysed 748 travellers to South and Southeast Asia with low or medium malaria transmission who had received SBET in a pre-travel consultation. Among the 100 febrile travellers carrying SBET during travel, only 14% took the correct measures (doctor visit or timely SBET administration) [23]. In the present study, the reasons for neither consulting nor performing the test were reasonable (diarrhoea and/or symptoms lasting a few hours only).

### Interest of travellers for RDTs

For a period of 2 years, the interest of travellers to use RDTs was studied more precisely. One out of ten (11%) travellers were not interested to travel with RDT because they did not want to prick themselves or intended to rely on on-site health facilities. When asked upon return, most travellers (90%) who had travelled with RDT were interested in taking RDT again for a future trip, mainly because they felt more secure. Those who did not want to mentioned that they would prefer to rely on on-site health facilities. These responses show that travellers have a good health judgment and that there is no ‘one size fits all’; every traveller chooses for themselves what they feel is most appropriate.

### RDT performance by travellers

Only one traveller could not perform the training run because of shaking hands. During the trip, all travellers except two (who then reacted appropriately) managed to perform the test without problem, probably thanks to the training run done before departure. An untrained febrile peer in Southern Sudan, who had been given an RDT by a study participant, was not able to perform the test despite the illustrated instruction leaflet. The trained traveller repeated the test on his colleague without difficulty and diagnosed malaria. This highlights the importance of performing a training run on oneself in good conditions before leaving, especially as it might be more challenging to perform the test once febrile (and especially amongst true malaria cases, as described by Jelinek [7]) or in a travelling environment.

In Jelinek’s prospective study assessing the use of dipstick RDTs among 98 European tourists consulting for fever in Kenya in 1998, 31% were unable to obtain a result. Among the 11 patients who had a microscopically confirmed malaria, 10 failed to diagnose themselves properly on-site [7]. In another study assessing self-testing for malaria in 153 symptomatic travellers returning from endemic areas, 18 did not want to be included because they were feeling too ill to concentrate, or they were not willing to prick themselves [8]. Lack of self-confidence can be overcome or at least minimized by training the traveller with performance of a practice run before departure, as done in this study. Reluctance to prick oneself was a reason not to provide the traveller with RDT. In this study, the most difficult step of RDT performance observed during the training run was blood aspiration with the pipette. For this reason the pipette included in the ICT Combo test^®^ was changed for an inverted cup. Two of the 462 travellers who left with a pipette-containing kit reported difficulty transferring blood to the device in the field. Collecting blood and manipulating blood transfer devices were also difficulties observed by Counihan et al. in assessing RDT use by community health workers with no medical background, which could be improved through training [10]. Therefore, choosing an RDT brand with an easy-to-use blood transfer device, such as an inverted cup, is crucial for use by a lay person, especially when self-testing [24].

### Interpretation of the RDT result

One traveller had difficulty reading the result because of a faint line appearing within 15 min. He took the SBET, as recommended in the written procedure in case of faint line. He mailed a picture of the test which was interpreted as a positive result. In a study assessing RDT use by community health workers in Zambia, Counihan et al. reported some difficulty reading faint positive test lines with the risk of interpreting as a negative result [10]. There is a positive correlation between line intensity and parasite density [11]; non-immune travellers at the beginning of a malaria episode have thus a higher probability of having a faint line than patients living in endemic areas [18]. Since the traveller is instructed here to repeat the test in case of persistent fever, the second test is expected to show a stronger line due to increasing parasitaemia.

### Action taken by travellers upon result of the test

Four travellers diagnosed themselves with malaria and all of them were able to start the SBET on time. They did not experience any adverse events. None of the negative travellers took SBET (but one received an antimalarial from a local clinic despite being tested negative again), which means that 47 unnecessary treatments could be avoided.

### Incidence of malaria and choice of travellers’ categories for RDT provision

The overall incidence of malaria (case definition: fever and positive RDT) in these travellers was 0.03 per 100 person-week (95% CI 0.0086–0.079), with an attack rate of 70/10,000 travellers. It was 0.07 per 100 person-week (185/10,000 travellers) in high and 0.02 per 100 person-week (52/10,000 travellers) in moderate- to low-risk areas where the risk of malaria is reported to be lower than 1/10,000 travellers [25]. The proportion of malaria was 4% among all fevers and 8% among fevers of more than a few hours. Even if the primary objective of this study was not to evaluate malaria incidence in travellers, the attack rate was much higher than expected; indeed, most of these travellers were visiting moderate- to low-risk endemic areas. These observations question the reliability of only estimating malaria incidence rate in travellers using denominators collected from tourist information at the macro-epidemiology level (national), as it has been done for Latin America and India for example—the incidence in UK travellers to Latin America was 0.8 per 10,000 visits with an average duration of stay of 18 days; in 2004 the estimated attack rate in French travellers to India was 0.01% [26–29]—and applying uniform malaria prevention strategies to all persons travelling in a specific country.

Bottieau et al. had already shown that *P. falciparum* malaria was more frequently diagnosed in expatriates (38%), VFR travellers (36%) and foreign visitors or migrants (26%) than in Western travellers (14%) [30]. A common trait amongst these groups is that they usually stay a longer period in the visited endemic region and are poorly compliant to preventive strategies against malaria [16]. They should thus certainly be considered at higher risk of exposure, even if they visit areas of low endemicity. Indeed, all positive cases in the present study were long-term travellers. As already suggested by some experts [11, 31], RDTs should be proposed to these categories of travellers, adding health care professionals and short-stay frequent travellers. Young children and pregnant women are at particular risk of developing severe malaria [1]; RDT could be also be used in these categories as an additional tool of chemoprophylaxis. Although at high risk, VFRs were not proposed RDTs because it was assumed that they were more aware of how to access appropriate health care.

The present strategy for malaria prevention in travellers is mainly based on the local level of malaria transmission derived from national statistics, and from the attack rate in travellers estimated through tourist information and imported malaria surveillance data [32–34]. The high malaria rates found in this cohort clearly shows that it is possible to better identify high risk groups during the pre-travel consultation; not only because they travel in high transmission regions, but also because they have specific personal and travel characteristics. So ideally, the malaria prevention recommendation should not only be geographically determined, but also tailored to an individual risk assessment. As this risk might be perceived and accepted differently from one traveller to the other, their preferences should also be taken into account [35]. In practice, high-risk category travellers for whom chemoprophylaxis is not indicated based on official recommendations, or those who are unwilling to take chemoprophylaxis, should be proposed RDTs with SBET, as this is probably a safer strategy than providing SBET alone. Adding RDTs to the usual SBET strategy also has the advantage of informing the traveller of the possibility of having *Plasmodium vivax* infection (rather than, or as well as *P. falciparum*), which may allow them to make more appropriate treatment decisions, such as including primaquine along with ACT. Another advantage of providing RDTs is that, when the result was negative, travellers could attend a health facility in search of an alternative cause for their fever: information that would likely also stimulate health workers to look for an alternative cause of the fever. This was suggested by the fact that travellers who subsequently attended on-site health facilities reported being diagnosed with gastroenteritis, amoebiasis, dengue, typhoid, pneumonia or urinary tract infection rather than empirically with malaria (except for one).

In addition, RDTs are now available on internet; only 4 out of the 8 RDTs sold on internet in 2011 had both good sensitivity and specificity [36]. It is thus certainly a better strategy to provide good quality RDTs during a pre-travel consultation than leaving travellers to purchase them through internet without guidance.

## Limitations

To prove that the provision of RDT and SBET is a perfectly safe strategy would have required a much larger sample size of travellers with fever. The same applies for demonstrating that the results of the RDTs were always well interpreted. Although travellers were asked to send pictures of the test performed, only 4 out of 58 were received, and 2/4 were from the positive cases. However, from the outcomes recorded, it is unlikely that malaria cases were missed, since none of the followed-up subjects developed malaria thereafter. Not all travellers meeting the criteria of the pre-defined categories were included, mostly due to time constraints of the consultation. This included travellers (especially families), who already required many other types of pre-travel advice and vaccines. This is one of the reasons why VFRs were underrepresented, albeit that they are not one of the classic pre-defined high-risk target groups.

## Conclusions

The vast majority of travellers provided with malaria RDTs used them according to the practical, oral and written instructions received during the pre-travel consultation. A fifth of febrile travellers neither performed the test nor consulted a health facility because the fever episode only lasted a few hours. Four travellers were able to self-diagnose and treat malaria adequately, which revealed a much higher incidence of malaria than expected. This strategy of RDT with SBET made the travellers feel more secure and allowed prompt treatment for malaria in high-risk groups. It has the additional advantage to avoid over-diagnosis of malaria and inappropriate on-site treatment. To ensure the success of this strategy, two key elements should be integrated in pre-travel consultations: (1) guiding travellers through a full training run to perform and interpret the RDT using their own blood; and (2) targeting travellers at higher risk of getting malaria. Thus, proposing pre-emptive RDTs in travel clinics may be a safe and effective option to better tailor malaria prevention to suit the individual needs and wishes of selected travellers, allowing them to make informed decisions on early treatment.

## Additional file

**Additional file 1.** Illustrated instruction leaflet.
