# Supplementary material for: Self-diagnosis of malaria by travellers: a cohort study on the use of malaria rapid diagnostic tests provided by a Swiss travel clinic
Source: Malar J. 2017 Oct 28;16:436. doi: 10.1186/s12936-017-2079-2 (PMC5660453; doi:10.1186/s12936-017-2079-2)
Supplement: Supplementary file 1 — Additional file 1. Illustrated instruction leaflet. [file 12936_2017_2079_MOESM1_ESM.docx]

**Additional File 1.**

(translated from the original procedure that was in French)

**How to perform a malaria Rapid Diagnostic Test during your trip to endemic areas ?**

# How to react upon the result of the malaria rapid diagnostic test during the trip

1. If you have fever during your trip, attend a medical center within 24 hours.
2. If you cannot attend a medical structure within 24 hours, perform a malaria Rapid Diagnostic Test (mRDT) following strictly the instructions underneath :
3. Control **C** Line missing 🡪 the test is invalid 🡪 perform a new test.
4. Control **C** Line present + **P.f** and / or **Pan** Test Lines present :
   positive test 🡪 take immediately the standby emergency treatment for malaria.
5. Control **C** Line present but no **P.f** et **Pan** line**:**
   negative test 🡪 do not take the standby emergency treatment for malaria.
6. In any case, independently of the result of RDT, it is necessary to consult a doctor as soon as possible (to evaluate the severity of malaria in case of positive RDT and find the cause of fever in case of negative RDT)

N.B: A negative mRDT doesn’t rule out malaria totally; if fever persists, it is imperative to attend a medical structure in order to repeat the mRDT after 24h and establish the diagnosis of fever.

# How to perform a malaria Rapid Diagnostic Test

## Material needed

- **Test** (Malaria Ag P.f/Pan) in the test packet

- **Sterile disposable lancet** to prick finger

- **Inverted cup tube** to collect blood

- **Alcohol swab** in the little bag

- **Buffer**

- **Watch or Clock** to calculate 15 minutes

- **Pencil** to write the date and hour of the test

## Specific steps

| 1. Open the plastic packet and take the test out.  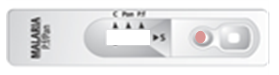 | 2. Write your name, day and hour at the back of the test. |
| --- | --- |
| 3. Firmly take the 4^th^ finger of the left hand. Disinfect it with the alcohol swab. Let the finger dry before pricking.  . | 1. Remove the plug of the lancet by turning it. Prepare the finger by massaging it upwards and prick it to obtain a drop of blood. If the drop is too small, make it bigger by squeezing the finger from bottom to top. |
| 1. Touch the drop of blood with the inverted cup of the transparent tube to collect a little portion of blood.   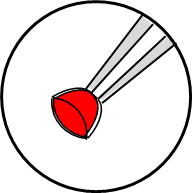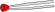 | 1. 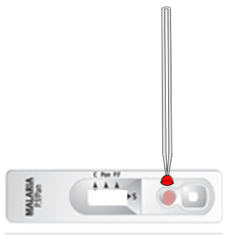Put the drop of blood in the round hole of the cassette by touching the absorbing pad at the base of the hole. Be aware of depositing the total amount of blood (if necessary gently tap the pad with the tube) |
| 7. Add 4 drops of the buffer solution in the big square hole of the cassette  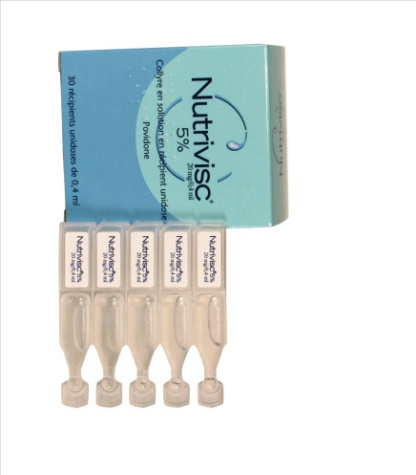  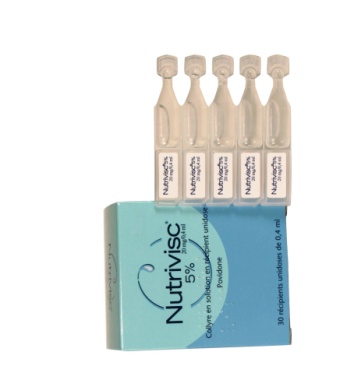  **4 drops**  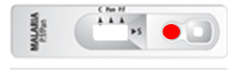 | 8. Wait minimum 15 minutes to read the result (max 30 minutes)    9. If possible, take a picture of the test test after 15 minutes for documentation. |

**Remark** : Don’t throw away the material with blood (lancet, inverted cup tube, alcohol swab) directly in the bin. Put the plug on the lancet again and keep the entire material wrapped in the plastic packet in a secure place (personal case…) in order to avoid that somebody gets in contact with your blood.

Interpretation of the result (big square window)

Control Line **C** : the line at the **C** level always has to appear.

- If it doesn’t, the test is **INVALID** ! You must then perform it again with a new test.

**Pan** and **P.f** test lines:

- If one or even 2 lines appear on the Pan or P.f side, the test is **POSITIVE** for malaria.

- If no line appears on the **Pan** or **P.f** side, the test is **NEGATIVE** for malaria.

| **POSITIVE test** for malaria  2 lines present: one side **C** and one side **P.f**  **C Pan P.f**  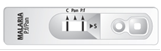 | **POSITIVE test** for malaria  2 lines present: one side **C** and one side **Pan**  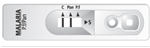  **C Pan P.f** | **POSITIVE test** for malaria  3 lines present: one side **C,**  one side **Pan,** one side **P.f**  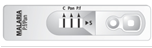  **C Pan P.f** |
| --- | --- | --- |
| **NEGATIVE test** for malaria  1 single line present on side **C**  **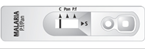**  **C Pan P.f** | **NEGATIVE test** for malaria  **C** line missing  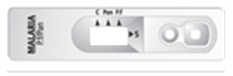  **C Pan P.f** |  |

Disclaimer : mTDR are performed under the responsability of the traveller and are sold to him/her on his/her request. The Travel Clinic is not responsible of potential consequences.
